# Fungal mucopyocele of the sphenoid sinus revealed by oculomotor and abducens nerve palsy: a case report

**DOI:** 10.1093/jscr/rjag256

**Published:** 2026-07-11

**Authors:** Mohammed Chehbouni, Salah-Eddine Youbi, Omar Oulghoul, Youssef Lakhdar, Othmane Benhoummad, Youssef Rochdi, Abdelaziz Raji

**Affiliations:** Faculty of Medicine and Pharmacy of Marrakech, Cadi Ayyad University, ENT Head and Neck Surgery Department, University Hospital Center Mohammed VI, Marrakech, Morocco; Faculty of Medicine and Pharmacy of Marrakech, Cadi Ayyad University, ENT Head and Neck Surgery Department, University Hospital Center Mohammed VI, Marrakech, Morocco; Faculty of Medicine and Pharmacy of Marrakech, Cadi Ayyad University, ENT Head and Neck Surgery Department, University Hospital Center Mohammed VI, Marrakech, Morocco; Faculty of Medicine and Pharmacy of Marrakech, Cadi Ayyad University, ENT Head and Neck Surgery Department, University Hospital Center Mohammed VI, Marrakech, Morocco; Faculty of Medicine and Pharmacy of Agadir, Ibn Zohr University, ENT Head and Neck Surgery Department, University Hospital Center Souss Massa Agadir, Morocco; Faculty of Medicine and Pharmacy of Marrakech, Cadi Ayyad University, ENT Head and Neck Surgery Department, University Hospital Center Mohammed VI, Marrakech, Morocco; Faculty of Medicine and Pharmacy of Marrakech, Cadi Ayyad University, ENT Head and Neck Surgery Department, University Hospital Center Mohammed VI, Marrakech, Morocco

**Keywords:** mucocele, sphenoid sinus, fungal infection, oculomotor nerve, abducens nerve, case report

## Abstract

Mucoceles of the sphenoid sinus are very rare accounting for 1% of all paranasal sinus mucoceles. Clinical symptoms are non-specific, extremely variable, and only occur when the mucocele impinges on surrounding structures. Secondary infection of a mucocele content is called mucopyocele and often result in rapid enlargement and acute onset of symptoms. The sphenoid sinus is closely related to important and vital structures accounting for the broad spectrum of clinical presentations. In this report we describe the case of a fungal mucopyocele of the sphenoid sinus, revealed by oculomotor and abducens nerve palsy, managed via endoscopic sphenoidotomy with complete postoperative resolution of ocular symptoms. Few reports in the recent literature have described a similar condition with insights on clinical aspects, surgical implications and functional outcomes.

## Introduction

Paranasal sinus mucoceles are benign cystic lesions, lined by respiratory epithelium and filled with mucus. They are capable of expansion and are locally invasive, leading to bony erosion and extension into adjacent structures. Mucoceles of the sphenoid sinus are very rare, accounting for 1% of all paranasal sinus mucoceles [1].

Clinical symptoms are non-specific, extremely variable, and only occur when the mucocele impinges on surrounding structures [2].

The sphenoid sinus is closely related to important structures such as optic nerve, internal carotid artery, cavernous sinus, oculomotor, abducens, and trochlear nerves, ophthalmic and maxillary divisions of the trigeminal nerve, pituitary gland, pterygopalatine fossa, and nasopharynx, accounting for the broad spectrum of clinical presentations.

Secondary infection of a mucocele is called mucopyocele. In addition, fungal infection in the sphenoid sinus is also a rare condition [3].

Here we report the case of a fungal mucopyocele of the sphenoid sinus revealed by oculomotor and abducens nerve palsy, managed by endoscopic sphenoidotomy via trans-ethmoid approach.

## Case report

A 52-year-old female patient presented to our Otolaryngology Head and Neck Surgery Department with an 8-month history of fluctuating right fronto-orbital headache, complicated 3 days before her admission by an acute onset of diplopia and dropping of the right superior eyelid. There were no other complaints, no loss of vision, no anosmia, nasal obstruction, or purulent nasal discharge, and no other neurological or ocular symptoms. She had no relevant medical history, no previous facial trauma, no history of endoscopic sinus or skull base surgery, chronic rhinosinusitis, or local radiation therapy.

On examination, she presented a blepharoptosis and ophthalmoplegia on the right side with restricted eye movements upon adduction, abduction, elevation, and depression, consistent with oculomotor and abducens nerve palsy (Fig. 1A). She had a normal visual acuity, no visual field defects, and normal fundoscopy. Furthermore, endoscopic examination of the nasal cavity revealed no abnormal findings. There were no other cranial nerve deficits, and the rest of the physical examination was unremarkable.

**Figure 1:**
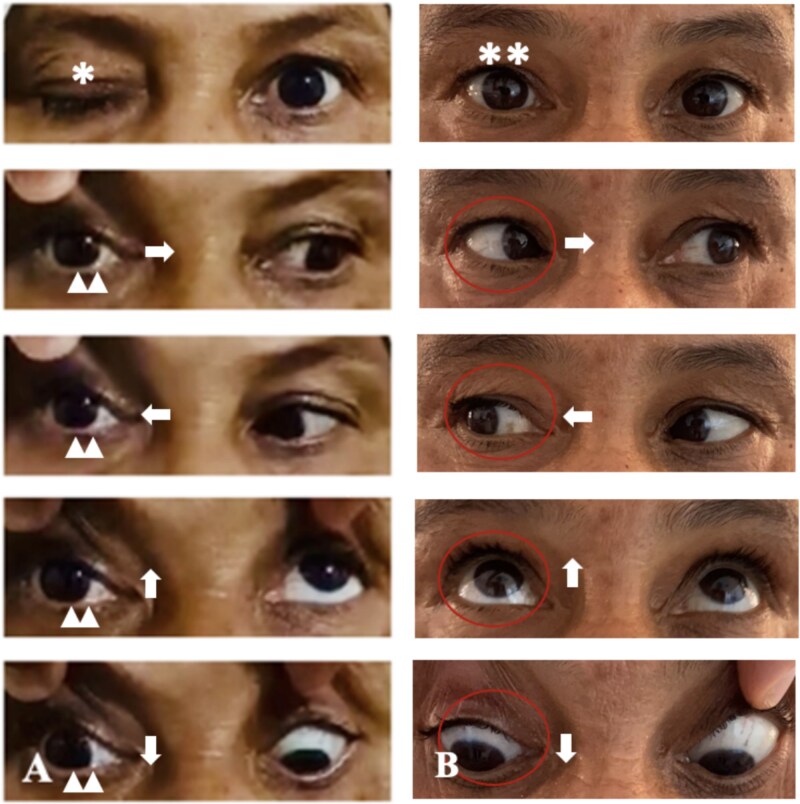
Pre-operative images (A) showing right blepharoptosis (^*^) and ophthalmoplegia (double arrowhead) with restricted eye movements upon adduction (right arrow), abduction (left arrow), elevation (up arrow), and depression (down arrow) consistent with third and sixth cranial nerve palsy. Post-operative images (B) showing complete resolution of blepharoptosis (^**^) and ophthalmoplegia.

Computed tomography (CT) scan showed a soft tissue mass in the right sphenoid sinus, causing marked expansion and erosion of its bony walls (Fig. 2). Magnetic resonance imaging (MRI) revealed a mass in the right sphenoid sinus, exhibiting high signal intensity on T_1_-weighted images, and intermediate signal intensity on T_2_-weighted images, along with marked enhancement after contrast injection. It was impinging on the right cavernous sinus, internal carotid artery, and orbital apex (Fig. 3). These imaging findings suggested a chronic, dehydrated mucocele within the sphenoid sinus, characterized by a high protein content.

**Figure 2:**
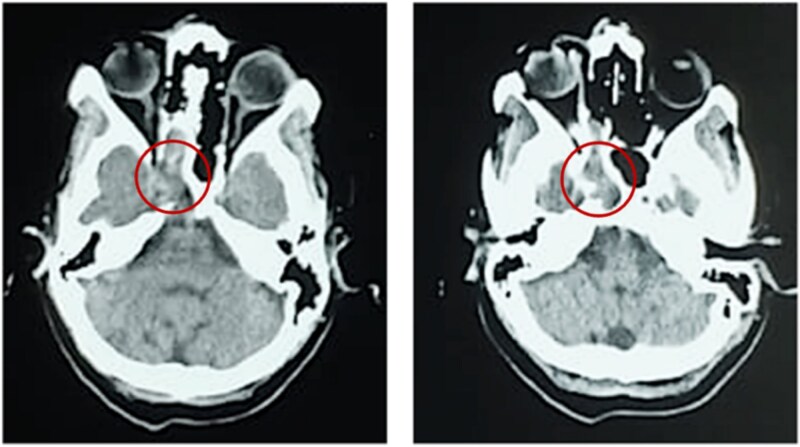
CT scan demonstrating a soft tissue mass in the right sphenoid sinus causing marked expansion and erosion of its bony walls.

**Figure 3:**
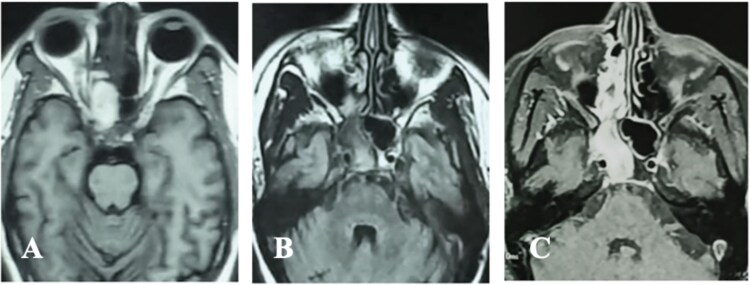
MRI illustrating a mass in the right sphenoid sinus, exhibiting high signal intensity on T1-weighted images (A), and intermediate signal intensity on T2-weighted images (B), along with marked enhancement after contrast injection (C).

The patient underwent surgery, during which we conducted a large sphenoidotomy via endoscopic trans-ethmoid approach, to marsupialize and decompress the mucocele. Intraoperatively, we discovered a yellow, thick, gluey material inside the sphenoid sinus, along with a brownish-green soft tissue mass suggestive of a fungus ball (Fig. 4). We cleared all the purulent mucous content of the mucocele and successfully removed the fungus ball from the sphenoid sinus.

**Figure 4:**
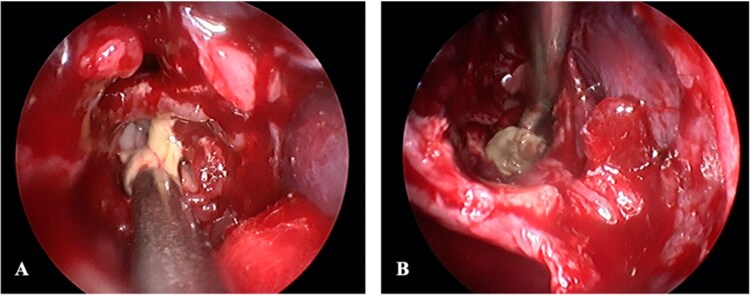
Operative view after posterior ethmoidectomy and sphenoidotomy, showing a yellow, thick, and gluey material (A), along with a brownish-green fungus ball (B) inside the sphenoid sinus.

No intraoperative or postoperative complications were noted. Mycology and histopathology confirmed the diagnosis of *Aspergillus* fungus ball. Postoperatively, we prescribed antibiotics, corticosteroids, and nasal saline irrigation. The patient did not receive any systemic antifungal therapy under the advice of the Infectious Diseases Department. Ocular symptom completely resolved after 4 weeks (Fig. 1B), and there was no evidence of recurrence after 2 years of follow-up.

## Discussion

Mucoceles of the sphenoid sinus are a very rare condition and represent only 1% of all paranasal sinus mucoceles [1, 4].

Clinical symptoms are non-specific, and often result in diagnosis delay. They depend on the direction of expansion of the mucocele [4–6], and only occurs when it causes bony destruction and impinges on surrounding structures. Most common symptoms include fronto-orbital headaches, visual loss, diplopia, blepharoptosis, and nasal symptoms such as anosmia, nasal obstruction, and nasal discharge [4, 5]. Secondary infection of a mucocele content is called mucopyocele and can result in rapid enlargement and acute onset of symptoms. It is often due to a bacterial infection, whereas mucopyocele of the sphenoid sinus of fungal origin is extremely rare, and only a few reports have been described in the literature.

Imaging plays an essential role in the early diagnosis of sphenoid sinus mucoceles, before onset of irreversible visual loss. CT scan indicates soft tissue density filling the sinus, responsible for expansion, thinning, and erosion of its bony walls [7]. MRI appearance is variable due to different signal intensities, depending on the mucocele’s water and protein content, and possible secondary infection [7]. MRI is also useful to study the extension of the mucocele and analyze surrounding structures. Differential diagnosis includes craniopharyngioma, cystic pituitary adenoma, and nasopharyngeal carcinoma, etc. [8]

Treatment consists of a sphenoidotomy with a large marsupialization to decompress and evacuate the mucocele, relieve symptoms, and avoid recurrence. Attempting to remove the entire mucosa of the sphenoid sinus can lead to inadvertent injury to vital structures, such as internal carotid artery and optic nerve, and is therefore not recommended [4]. Endoscopic approach is the modality of choice and offers many advantages.

In this report, we describe the case of fungal mucopyocele of the sphenoid sinus, revealed by oculomotor and abducens nerve palsy, managed via endoscopic sphenoidotomy. Intraoperative findings were consistent with a fungus ball inside a sphenoid sinus mucopyocele. Few reports in the recent literature have described a similar condition with insights on clinical aspects, surgical implications, and functional outcomes.

Lee *et al.* [3] described three cases of fungal mucopyocele with a fungus ball inside the sphenoid sinus, successfully managed via endoscopic sphenoidotomy, with good postoperative results and no signs of recurrence upon follow-up.

Naito *et al.* [9] reported a case of sphenoid sinus mucocele complicated by abducens nerve palsy, also managed by endoscopic sphenoidotomy.

Other authors reported sphenoid sinus mucoceles revealed by blepharoptosis and ophthalmoplegia due to oculomotor nerve involvement, also managed successfully via endoscopic approach [2, 6, 10].

Our case shows that chronic headaches with recent onset of ocular symptoms can result from a sphenoid sinus mucocele. Imaging is crucial for diagnosis and should help the surgeon rule out other causes of ophthalmoplegia that might require adequate treatment. Endoscopic sphenoidotomy is a safe and reliable treatment option that allows decompression of the mucocele and rapid resolution of symptoms.

## Conclusion

Mucoceles of the sphenoid sinus are very rare.

Secondary infection of the mucocele content can result in rapid enlargement and acute onset of symptoms due to compression of important and vital surrounding structures.

Clinical symptoms, although non-specific, should alert the surgeon to a potential locally invasive process.

Imaging is crucial for early diagnosis and management to prevent further extension and onset of irreversible sequelae such as permanent visual loss.

Endoscopic approach offers many advantages and represents the treatment of choice.
